# Critical Time Intervals in Door-to-Balloon Time Linked to One-Year Mortality in ST-Elevation Myocardial Infarction

**DOI:** 10.5811/westjem.20779

**Published:** 2025-01-30

**Authors:** Shin-Ho Tsai, Yu-Ting Hsiao, Ya-Ni Yeh, Jih-Chun Lin, Shi-Quan Zhang, Ming-Jen Tsai

**Affiliations:** Ditmanson Medical Foundation Chia-Yi Christian Hospital, Department of Emergency Medicine, Chiayi City, Taiwan

## Abstract

**Background:**

Timely activation of primary percutaneous coronary intervention (PCI) is crucial for patients with ST-segment elevation myocardial infarction (STEMI). Door-to-balloon (DTB) time, representing the duration from patient arrival to balloon inflation, is critical for prognosis. However, the specific time segment within the DTB that is most associated with long-term mortality remains unclear. In this study we aimed to identify the target time segment within the DTB that is most associated with one-year mortality in STEMI patients.

**Methods:**

We conducted a retrospective cohort study at a tertiary teaching hospital. All patients diagnosed with STEMI and activated for primary PCI from the emergency department were identified between January 2013–December 2021. Patient demographics, medical history, triage information, electrocardiogram, troponin-I levels, and coronary angiography reports were obtained. We divided the DTB time into door-to-electrocardiogram (ECG), ECG-to-cardiac catheterization laboratory (cath lab) activation, activation-to-cath lab arrival, and cath lab arrival-to-balloon time. We used Kaplan-Meier survival analysis and multivariable Cox proportional hazards models to determine the independent effects of these time intervals on the risk of one-year mortality.

**Results:**

A total of 732 STEMI patients were included. Kaplan-Meier analysis revealed that delayed door-to-ECG time (>10 min) and cath lab arrival-to-balloon time (>30 min) were associated with a higher risk of one-year mortality (log-rank test, *P* < .001 and *P* = 0.01, respectively). In the multivariable Cox models, door-to-ECG time was a significant predictor for one-year mortality, whether it was analyzed as a dichotomized (>10 min vs ≤10 min) or a continuous variable. The corresponding adjusted hazard ratios (aHR) were 2.81 (95% confidence interval [CI] 1.42–5.55) for the dichotomized analysis, and 1.03 (95% CI 1.00–1.06) per minute increase, respectively. Cath lab arrival-to-balloon time also showed an independent effect on one-year mortality when analyzed as a continuous variable, with an aHR of 1.02 (95% CI 1.00–1.04) per minute increase. However, ECG-to-cath lab activation and activation-to-cath lab arrival times did not show a significant association with the risk of one-year mortality.

**Conclusion:**

Within the door-to-balloon interval, the time from door-to-ECG completion is particularly crucial for one-year survival after STEMI, while cath lab arrival-to-balloon inflation may also be relevant.

Population Health Research Capsule What do we already know about this issue?

*Timely percutaneous coronary intervention is essential for patients with ST-elevation myocardial infarction (STEMI); reducing door-to-balloon (DTB) time improves survival outcomes.*

What was the research question?

*Which interval within the DTB time is most associated with 1-year mortality in STEMI patients?*

What was the major finding of the study?

*The time from door-to-ECG completion within the DTB interval is particularly important for 1-year mortality in STEMI patients.*

How does this improve population health?

*Identifying key intervals within DTB time associated with long-term mortality in STEMI patients supports the development of targeted improvement strategies.*

## INTRODUCTION

Primary percutaneous coronary intervention (PCI) stands as the cornerstone therapy for patients experiencing ST-segment elevation myocardial infarction (STEMI).^1^ ^–^ ^3^ The prompt activation of primary PCI upon a STEMI patient’s arrival at the emergency department (ED) is crucial for achieving coronary artery reperfusion.^1^ ^–^ ^3^ The door-to-balloon (DTB) time, representing the interval from the patient’s ED arrival to the inflation of a balloon within the occluded coronary artery, serves as a pivotal metric in this process.^1^ ^,^ ^3^ Prolonged DTB times have consistently been associated with an elevated risk of short-term mortality and major adverse cardiac events.^4^ ^–^ ^6^ Consequently, DTB time serves as a quality indicator for assessing the performance of a PCI-capable hospital.^1^ ^,^ ^7^

Within the DTB time, several time segments can be delineated, including door-to-electrocardiogram (ECG), ECG-to-catheterization laboratory (cath lab) activation, activation-to-cath lab arrival, cath lab arrival-to-needle insertion, and needle insertion-to-balloon inflation time. Delays in any of these time segments may lead to prolonged DTB time.^7^ ^–^ ^9^

Previous studies have explored the relationship between DTB and short-term mortality, such as in-hospital death or 30-day mortality.^5^ ^,^ ^6^ However, the impact of DTB time on long-term mortality and which specific time segment within the DTB is mostly associated with long-term outcome remain unclear. To aid in the development of improvement strategies, our goal in this study was to determine the target period within the DTB that is most associated with one-year mortality in STEMI patients.

## METHODS

### Study Design, Setting, and Participants

We conducted a retrospective cohort study at Ditmanson Medical Foundation Chia-Yi Christian Hospital, a 1,000-bed tertiary teaching hospital in an urban city of Taiwan. The hospital’s emergency department (ED) handles approximately 80,000 patient visits annually. Designated as an accredited, advanced emergency-responsibility hospital in Taiwan since 2013, it undergoes regular evaluations to ensure compliance with STEMI emergency management standards. Key objectives include providing 24/7 emergency cardiac catheterization services, ensuring that over 80% of STEMI patients receive an ECG examination within 10 minutes of ED arrival, initiating dual antiplatelet therapy for at least 80% of STEMI patients in the ED before primary PCI, and achieving DTB times of under 90 minutes for over 75% of STEMI patients. Consequently, a protocol for managing STEMI patients in the ED has been implemented (Supplementary Figure 1).

**Figure 1.**
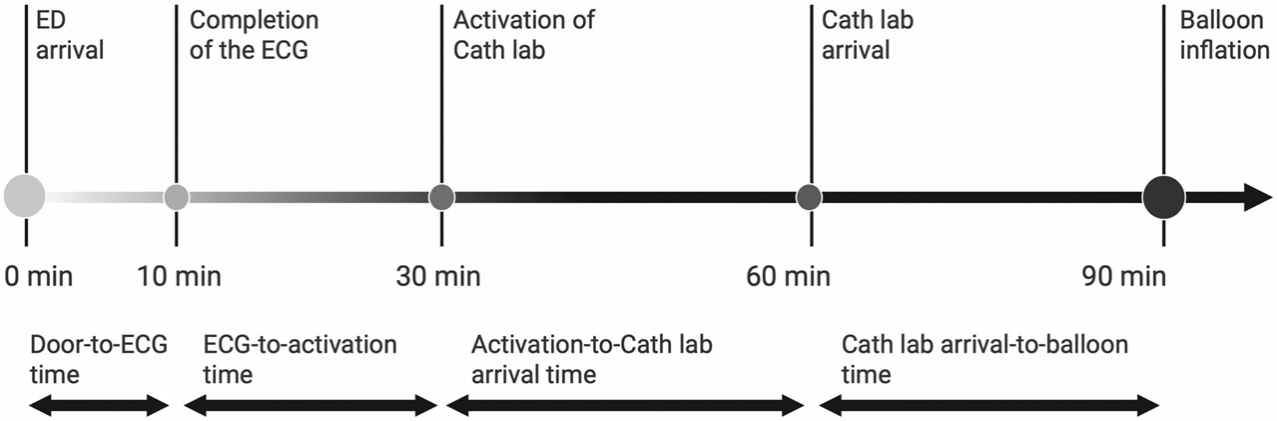
Time segments within the door-to-balloon time. *Cath lab*, catheterization laboratory; *ECG*, electrocardiogram; *ED*, emergency department.

Upon arrival, immediate ECG is performed for patients with any cardiopulmonary-related symptoms in triage and promptly reviewed by an emergency physician. If STEMI is diagnosed, a loading dose of dual antiplatelet therapy and anticoagulants is administered, and the cardiologist is immediately consulted. The cardiologist activates primary PCI after assessment. Once the cath lab is prepared, the patient is transferred for primary PCI as expeditiously as possible.

All patients diagnosed with STEMI and activated for primary PCI from the ED are included in the hospital-based STEMI registry, where data was prospectively gathered for quality improvement. We identified patients hospitalized for STEMI between January 1, 2013–December 31, 2021, from the STEMI registry. Factors potentially influencing STEMI outcomes and DTB time, such as demographic data (age, sex, body mass index).^10^ ^,^ ^11^ time of ED arrival,^12^ weekend visit,^13^ visit during the COVID-19 pandemic,^4^ ^,^ ^15^ mode of transportation to the hospital,^16^ triage level,^17^ initial troponin-I levels,^18^ comorbidities,^11^ findings of ECG and coronary angiography,^10^ ^,^ ^11^ and duration of hospitalization, were collected from the registry and electronic health records.

Various time points from ED arrival to balloon inflation were identified, including the time of completion of the first ECG, activation of the cath lab, arrival in the cath lab, and balloon inflation. We defined door-to-ECG time as the duration from ED arrival to completion of the first ECG, ECG-to-activation time as the duration from ECG completion to activation of the cath lab, activation-to-cath lab-arrival time as the duration from cath lab activation to the patient’s arrival in the cath lab, and cath lab-arrival-to-balloon time as the duration from the patient’s arrival in the cath lab to balloon inflation (Figure 1).^1^ ^,^ ^3^ ^,^ ^7^ Delays in different time intervals within the DTB were defined as follows: door-to-ECG time >10 min; ECG-to-activation time >20 min; activation-to-cath lab arrival time >30 min; cath lab arrival-to-balloon time >30 min; and DTB time >90 min.^1^ ^,^ ^5^

### Outcome Measurement

The primary outcome assessed in this study was all-cause mortality within one year after admission for STEMI. All STEMI patients were followed up for at least one year from the date of admission to assess mortality. Mortality timing was accurately determined by cross-referencing study patients with the National Cause of Death Registry from the Taiwan National Health Insurance database, which documents the time and cause of death for all deceased individuals in Taiwan.^19^ The last follow-up date was December 31, 2022. Since Taiwan National Health Insurance is a compulsory, single-payer healthcare system covering nearly 99.8% of the population, theoretically all enrolled patients who pass away are recorded in the National Cause of Death Registry.^19^ Thus, unless a patient withdraws from the insurance systems, all included patients can be tracked either until the last follow-up date or their date of death. Patients with out-of-hospital cardiac arrest (OHCA) and those with missing data were excluded from the analysis.

This was a health record review study in which we followed the methodological criteria for health record review studies proposed by Woster et al.^20^ After identifying STEMI patients from the hospital-based STEMI registry, we used a pre-designed form with defined variables to record patients’ data. Four trained emergency residents and nurses reviewed the electronic health records and input data into the form. Regular meetings were held to ensure the correctness of data collection, and a supervisor randomly audited the accuracy of the data collected. The data abstractors were not aware of the study’s hypothesis and were informed only that they were helping to establish a STEMI database for research purposes. The study protocol received approval from the Institutional Review Board of Ditmanson Medical Foundation Chia-Yi Christian Hospital (approval number: CYCH-IRB 2024010), with an exemption from informed consent owing to the retrospective nature of the study.

### Statistical Analysis

We compared data from the included STEMI patients between two groups: those with and without one-year mortality. Continuous variables were expressed as mean ± standard deviation or median (interquartile range) and assessed between groups using the Student *t*-test or Mann-Whitney U test, respectively, based on data distribution. We present categorical variables as number (percentage) and assessed them using chi-square test. The mortality rate was expressed as events per 100 person-years. To identify the target time segment within the DTB most associated with one-year mortality, we employed Kaplan-Meier survival analysis. Survival curves were plotted for patients stratified into delay vs non-delay groups across different time intervals within the DTB, with differences assessed using log-rank tests. We used univariable Cox proportional hazards models to assess the association between each variable and one-year mortality. Time intervals within the DTB were treated as either dichotomized (delay or non-delay groups) or continuous variables. We further analyzed variables demonstrating a *P*-value of less than 0.1 in the univariable analysis in a multivariable Cox model employing forward variable selection (set at *P* < 0.05 for addition to the model) to determine their independent effect on the risk of one-year mortality. The Schoenfeld test was subsequently used to verify the assumption of proportional hazards.

### Sensitivity Analysis

We conducted additional sensitivity analyses to examine the association between various time intervals within the DTB and short-term mortality outcomes, such as in-hospital and 30-day mortality, as well as one-year mortality. These analyses used multivariable logistic regression with a forward stepwise Wald test. The variables included in these analyses were the same as those in the multivariable Cox analysis. The time intervals within the DTB were incorporated into the models separately and were treated as either dichotomized or continuous variables. Furthermore, considering the extended recruitment period of this study (nine years), we conducted another sensitivity analysis to control for potential confounding factors across different time periods. In addition to adjusting for associated variables, we performed a multivariable Cox model including the year of patient recruitment as a covariate. Finally, a sensitivity analysis using a multivariable Cox model was conducted to evaluate the relationship between DTB time intervals and cardiovascular-related one-year mortality. We performed statistical analyses using Stata 17.0 (StataCorp, College Station, TX), with statistical significance set at two-tailed <0.05.

## RESULTS

**Figure 2.**
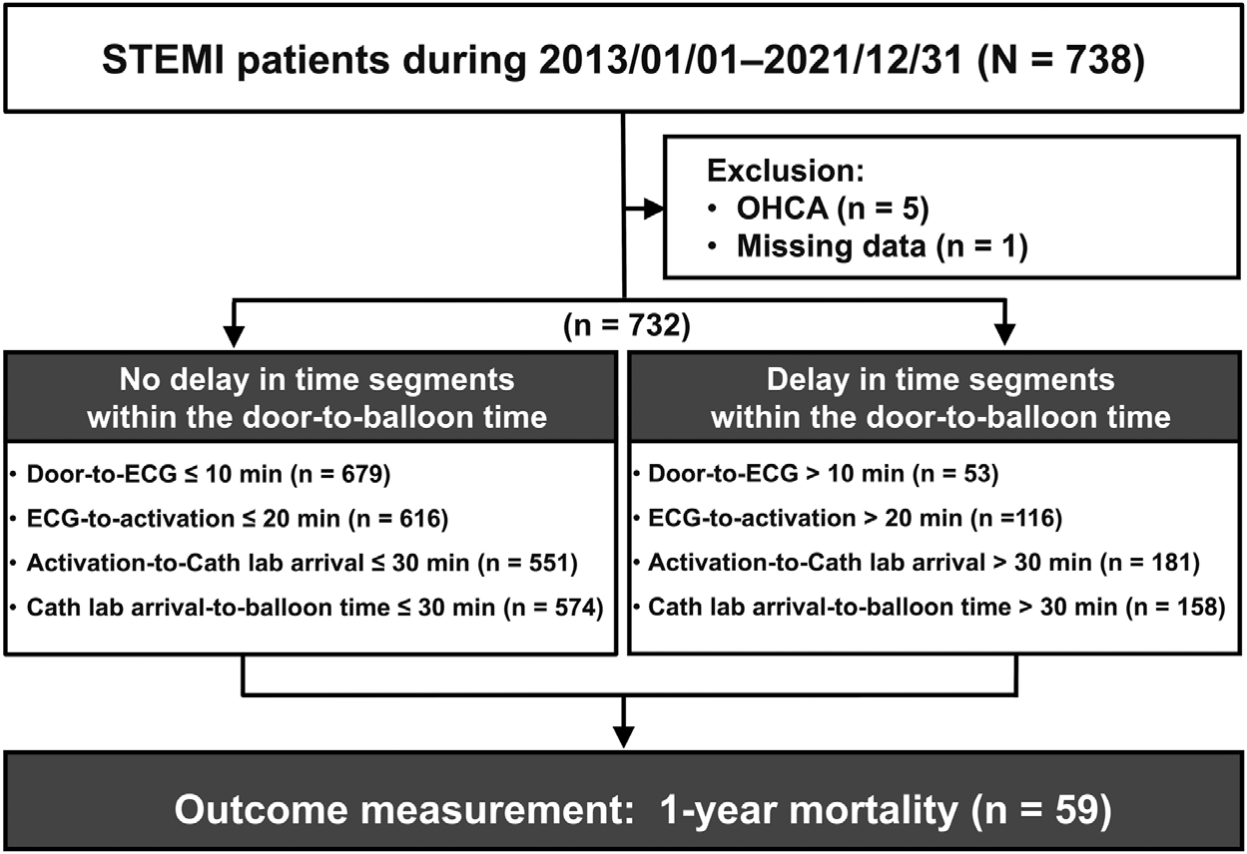
Flowchart of the patients included in the study. *ECG*, electrocardiogram; *OHCA*, out-of-hospital cardiac arrest; *STEMI*, ST-segment elevation myocardial infarction.

During the study period, 738 patients with STEMI were identified. After excluding those with OHCA or missing data, 732 patients were finally included. Among them, 59 patients died within one year after STEMI (Figure 2), with 37 deaths attributed to cardiovascular-related causes. The overall mortality rate was 9.05 per 100 person-years (95% confidence interval [CI], 7.02–11.69).

Table 1 presents the characteristics of patients with and without mortality within one year after STEMI. Patients who died within one year after STEMI were older (73.2 ± 13.7 vs 61.0 ± 12.6 years, *P* < .001) and had a higher proportion of females (35.59% vs 14.56%, *P* < .001). They were more likely to be transported to the hospital by ambulance (40.68% vs 24.37%, *P* = 0.006) and had higher triage acuity (triage level 1: 32.2% vs. 8.92%, *P* < .001) and initial troponin-I levels (1.72 [0.12–8.52] vs 0.13 [0.02–2.40) nanograms per milliliter, *P* < .001]. They were also more likely to have diabetes mellitus (54.24% vs 38.04%, *P* = 0.02), hypertension (76.27% vs 63.30%, *P* = 0.05), cerebrovascular accident (20.34% vs. 6.39%, *P* < .001), and chronic kidney disease (20.34% vs 6.69%, *P* < .001), while being less likely to have hyperlipidemia (27.12% vs 63.30%, *P* < .001). Additionally, they had longer hospitalization durations (6 [3–14] vs 5 [4–6] days, *P* = 0.02) and longer door-to-ECG (7 [5–11] vs 4 [3–6] min, *P* < .001], cath lab arrival-to-balloon (25 [17–40] vs 22 [16–28] min, *P* = 0.02), and DTB times (74 [56–88] vs 64 [52–75] min, *P* < .001]. Moreover, a higher proportion of patients had DTB time longer than 90 min (22.03% vs 8.82%, *P* < .001).

**Table 1.** Characteristics of patients with and without one-year mortality after admission for STEMI*.

Characteristics	1-year survival (N = 673)	1-year mortality (N = 59)	*P-value*
Age (year)	61.0 ± 12.6	73.2 ± 13.7	<.001
BMI	25.1 (23.0–27.8)	24.1 (21.1–27.5)	0.08
Female sex	98 (14.56)	21 (35.59)	<.001
Patient arrival time			
Day shift	294 (43.69)	33 (55.93)	0.19
Evening shift	251 (37.30)	17 (28.81)	
Night shift	128 (19.02)	9 (15.25)	
Weekend visit	197 (29.27)	18 (30.51)	0.84
During COVID-19 pandemic (2020–2021)	143 (21.25)	8 (13.56)	0.16
Ambulance-transported patient	164 (24.37)	24 (40.68)	0.006
Triage level			
1	60 (8.92)	19 (32.20)	<.001
2	553 (82.17)	38 (64.41)	
3	60 (8.92)	2 (3.39)	
Laboratory test			
Troponin-I (ng/mL)	0.13 (0.02–2.40)	1.72 (0.12–8.52)	<.001
Medical history			
Diabetes mellitus	256 (38.04)	32 (54.24)	0.02
Hypertension	426 (63.30)	45 (76.27)	0.05
Hyperlipidemia	426 (63.30)	16 (27.12)	<.001
Cerebrovascular accident	43 (6.39)	12 (20.34)	<.001
Chronic kidney disease	45 (6.69)	12 (20.34)	<.001
Coronary artery disease	92 (13.67)	9 (15.25)	0.74
COPD	18 (2.68)	4 (6.78)	0.08
PAOD	8 (1.19)	1 (1.70)	0.53
Smoking	410 (60.92)	29 (49.15)	0.08
ECG report			
Anterior STEMI	305 (45.32)	31 (52.54)	0.29
Inferior STEMI	340 (50.67)	25 (42.37)	0.22
Lateral STEMI	23 (3.43)	2 (3.39)	1.00
Posterior STEMI	12 (1.79)	0 (0.00)	0.61
Numbers of vessel disease	2 (1–3)	2 (2–3)	0.06
Findings of coronary angiography			
1 vessel disease	206 (30.66)	14 (23.73)	0.23
2 vessels disease	244 (36.31)	18 (30.51)	
3 vessels disease	220 (32.74)	27 (45.76)	
Duration of hospitalization (day)	5 (4–6)	6 (3–14)	0.02
Time interval			
Door-to-ECG (min)	4 (3–6)	7 (5–11)	<.001
ECG-to-activation (min)	10 (6–15)	11 (8–19)	0.13
Activation-to-cath lab arrival (min)	23 (16–30)	22 (16–30)	0.94
Cath lab arrival-to-balloon (min)	22 (16–28)	25 (17–40)	0.02
Door-to-balloon (min)	64 (52–75)	74 (56–88)	<.001
Door-to-balloon > 90 min	58 (8.62)	13 (22.03)	<.001

Data are presented as n (%), mean ± SD, or median (interquartile range).

*BMI*, body mass index; *COPD*, chronic obstructive pulmonary disease; *ECG*, electrocardiography; *ng/mL*, nanograms per milliliter; *PAOD*, peripheral arterial occlusion disease; *STEMI, ST-segment elevation myocardial infarction.

**Figure 3.**
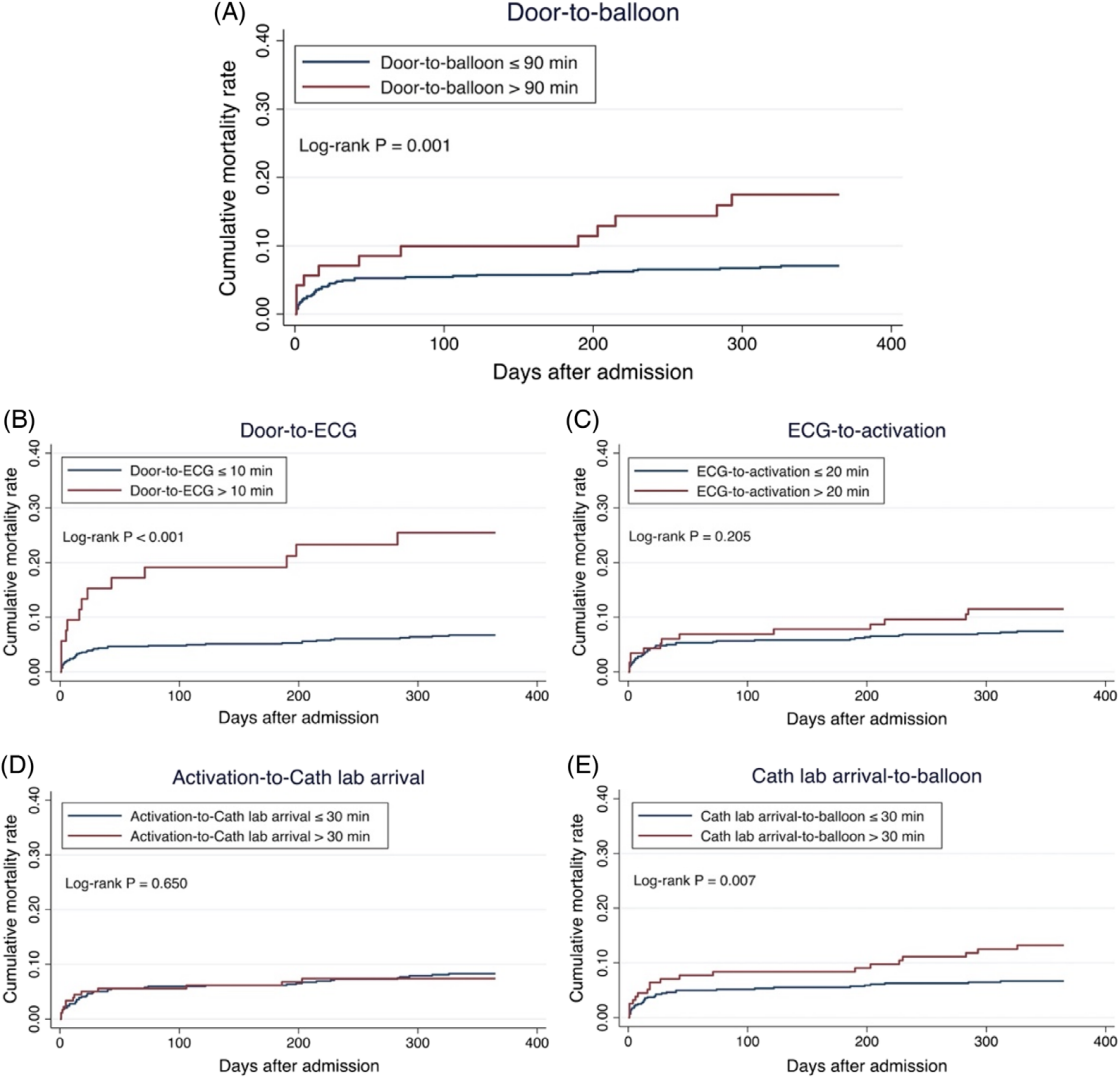
Kaplan-Meier curves illustrating cumulative mortality rates within a one-year follow-up period after STEMI* admission for delay and non-delay groups in door-to-balloon (A), door-to-ECG (B), ECG-to-activation (C), activation-to-cath lab arrival (D), and cath lab arrival-to-balloon (E) times. *STEMI, ST-elevation myocardial infarction.

Figure 3 shows the Kaplan-Meier curves for mortality after admission for STEMI. We analyzed the mortality probability between two groups based on the DTB time (Figure 3A). The cumulative mortality rate was significantly higher in the delayed DTB group (DTB > 90 min) compared to the non-delayed group (DTB ≤ 90 min) during the one-year follow-up period (long-rank test, *P* < .001). Next, we separately analyzed for the different time segments within the DTB. Patients with delayed door-to-ECG (Figure 3B) and cath lab arrival-to-balloon times (Figure 3E) had a higher mortality risk than their non-delayed counterparts (*P* < .001 and P = 0.007, respectively). However, no significant difference was observed between patients with and without delays in ECG-to-cath lab activation (Figure 3C) and activation-to-cath lab arrival (Figure 3D).

In the univariable Cox analyses (Table 2), an increase in age, female sex, ambulance-transported patients, higher troponin-I levels, a medical history of diabetes, hypertension, cerebrovascular accident, or chronic kidney disease, and each additional day of hospitalization were associated with a higher risk of one-year mortality. Additionally, patients with a lower triage level and hyperlipidemia had a lower risk of one-year mortality. Moreover, door-to-ECG and cath lab arrival-to-balloon times were significantly associated with a higher risk of onw-year mortality after STEMI.

**Table 2.** Univariable Cox models for predicting one-year mortality after STEMI* admission.

Characteristics	Univariable unadjusted HR (95% CI)	*P-value*
Age (year)	1.08 (1.05–1.10)	<.001
BMI	0.94 (0.88–1.01)	0.11
Female sex	2.95 (1.73–5.03)	<.001
Patient arrival time		
Day shift	Reference	
Evening shift	0.63 (0.35–1.13)	0.12
Night shift	0.63 (0.30–1.31)	0.21
Weekend visit	1.06 (0.61–1.84)	0.85
During COVID-19 pandemic (2020–2021)	0.63 (0.30–1.33)	0.23
Ambulance-transported patient	2.06 (1.23–3.47)	0.006
Triage level		
1	Reference	
2	0.24 (0.14–0.41)	<.001
3	0.11 (0.03–0.48)	0.003
Laboratory test		
Troponin-I (ng/mL)	1.01 (1.01–1.02)	<.001
Medical history		
Diabetes mellitus	1.85 (1.11–3.08)	0.02
Hypertension	1.84 (1.01–3.35)	0.05
Hyperlipidemia	0.23 (0.13–0.40)	<.001
Cerebrovascular accident	3.35 (1.78–6.31)	<.001
Chronic kidney disease	3.13 (1.66–5.91)	<.001
Coronary artery disease	1.17 (0.58–2.38)	0.66
COPD	2.42 (0.88–6.69)	0.09
PAOD	1.35 (0.19–9.76)	0.77
Smoking	0.63 (0.38–1.05)	0.07
ECG report		
Anterior STEMI	1.36 (0.81–2.26)	0.24
Inferior STEMI	0.71 (0.43–1.20)	0.20
Lateral STEMI	0.98 (0.24–4.00)	0.97
Posterior STEMI	4.53E-15 (0–∞)	1.00
Numbers of vessel disease	1.37 (0.99–1.90)	0.06
Duration of hospitalization (day)	1.03 (1.02–1.05)	<.001
Time interval		
Door-to-ECG >10 min	4.82 (2.68–8.66)	<.001
ECG-to-activation > 20 min	1.48 (0.80–2.75)	0.21
Activation-to-cath lab arrival > 30 min	0.87 (0.47–1.61)	0.65
Cath lab arrival-to-balloon time >30 min	2.05 (1.20–3.49)	0.008
Time interval		
Door-to-ECG time (min)	1.02 (1.00–1.04)	0.03
ECG-to-activation time (min)	1.00 (0.99–1.01)	0.91
Activation-to-cath lab arrival time (min)	1.01 (0.99–1.03)	0.50
Cath lab arrival-to-balloon time (min)	1.03 (1.02–1.05)	<.001

*BMI*, body mass index; *COPD*, chronic obstructive pulmonary disease; *CI*, confidence interval; *ECG*, electrocardiography;

*HR*, hazard ratio; *PAOD*, peripheral arterial occlusion disease; *STEMI, ST-segment elevation myocardial infarction.

Table 3 presents the results of multivariable Cox analyses. In model 1, we analyzed the time intervals as dichotomized variables (delay vs non-delay groups). After adjusting for associated factors, a delayed door-to-ECG time (>10 min) remained an independent predictor of one-year mortality, with an adjusted hazard ratio (HR) of 2.81 (95% CI 1.42–5.55). In model 2, the time intervals were analyzed as continuous variables. We found that each minute increase in door-to-ECG time (adjusted HR, 1.03; 95% CI 1.00–1.06) and cath lab arrival-to-balloon time (adjusted HR, 1.02; 95% CI 1.00–1.04) were independently associated with one-year mortality. Furthermore, age, triage level, initial troponin-I levels, and a history of diabetes mellitus and hyperlipidemia were independent predictors of one-year mortality (Table 3). The Schoenfeld test yielded *P*-values of 0.65 and 0.43 for models 1 and 2, respectively, indicating no violation of the proportional hazards assumption for the included covariates.

**Table 3.** Multivariable Cox models for predicting one-year mortality after STEMI^*^ admission.

Characteristics^**^	Model 1 adjusted HR (95% CI)	*P-value*	Model 2 adjusted HR (95% CI)	*P-value*
Age (year)	1.06 (1.03–1.08)	<.001	1.06 (1.03–1.08)	<.001
Triage level				
1	Reference		Reference	
2	0.35 (0.19–0.62)	<.001	0.33 (0.18–0.59)	<.001
3	0.14 (0.03–0.62)	0.01	0.12 (0.02–0.66)	0.02
Troponin-I (ng/mL)	1.01 (1.00–1.01)	0.05	1.01 (1.00–1.01)	0.02
Diabetes mellitus	1.77 (1.01–3.10)	0.05	1.90 (1.08–3.32)	0.03
Hyperlipidemia	0.31 (0.17–0.58)	<.001	0.31 (0.16–0.57)	<.001
Time interval				
Door-to-ECG >10 min	2.81 (1.42–5.55)	0.003		
Cath lab arrival-to-balloon >30 min	-			
Time interval				
Door-to-ECG time (min)			1.03 (1.00–1.06)	0.04
Cath lab arrival-to-balloon time (min)			1.02 (1.00–1.04)	0.02

** The variables included in the multivariable Cox model with forward selection analysis were age, sex, ambulance-transported patient, triage level, troponin I, diabetes mellitus, hypertension, hyperlipidemia, cerebrovascular accident, chronic kidney disease, numbers of vessel disease, duration of hospitalization, door-to-ECG time, and cath lab arrival-to-balloon time. The characteristics presented in the table represent the variables that were ultimately selected for inclusion in the Cox models.

*ECG*, electrocardiography; *HR*, hazard ratio; *ng/mL*, nanograms per milliliter; *STEMI, ST-segment elevation myocardial infarction.

Supplementary Table 1 presents the sensitivity analysis conducted using logistic regression for the outcomes of in-hospital, 30-day and one-year mortality. After adjusting for associated factors, door-to-ECG time was consistently identified as an independent predictor for in-hospital, 30-day, and one-year mortality, regardless of whether it was analyzed as dichotomized or continuous variables. Moreover, cath lab-arrival-to balloon time was identified as an independent predictor for the one-year mortality outcome when it was analyzed as a continuous variable. Supplementary Table 2 displays another sensitivity analysis that included the year of patient recruitment as a covariate to address potential confounding factors across different time periods. The results were similar to the main analysis. Supplementary Table 3 shows the results of a sensitivity analysis focused on one-year cardiovascular-related mortality, which were consistent with the main findings.

## DISCUSSION

In this study, we explored specific time segments within the DTB interval associated with one-year mortality in patients with STEMI. Our findings revealed that the duration from door to ECG completion is particularly associated with one-year mortality, while cath lab arrival-to-balloon inflation may also be relevant. However, the intervals from ECG completion to cath lab activation and from activation-to-cath lab arrival were not significantly associated with one-year mortality. Additionally, age, triage acuity level, initial troponin-I levels, and a history of diabetes mellitus and hyperlipidemia were identified as independent predictors for one-year mortality in STEMI patients.

We observed that a delay in door-to-ECG time (>10 min) was associated with a 2.81-fold increased risk of mortality within one year compared to those without delay. Each minute delay in ECG acquisition may increase the risk of mortality by 3% within one year after STEMI (Table 3). This highlights the importance of early ECG acquisition for the long-term prognosis of STEMI. During the treatment course for patients with STEMI, therapeutic interventions, such as early administration of dual antiplatelet agents, anticoagulants, and vigilant monitoring, are initiated upon STEMI diagnosis, prior to angiographic assessment (Supplementary Figure 1). Current guidelines recommend early platelet inhibition as a fundamental component of pharmacologic treatment in the early stages of STEMI, with expected benefits including enhanced platelet inhibition after primary PCI and a lower incidence of stent thrombosis.^3^ ^,^ ^21^ ^,^ ^22^

A recent systematic review and meta-analysis additionally also shows the importance of using upstream anticoagulation before PCI, which is associated with a lower 30-day mortality risk, a lower incidence of in-hospital cardiogenic shock, and improved reperfusion of the infarct-related artery.^23^ Early ECG completion enables prompt diagnosis of STEMI and timely initiation of antiplatelet agents, anticoagulants, and intensive care, which are crucial for achieving coronary artery reperfusion, preventing thrombosis following primary PCI, and minimizing myocardial damage post-infarction.^22^ ^,^ ^23^ Consequently, as observed in this study, not only is short-term mortality improved, but long-term mortality as well. Our findings also support the importance of prehospital ECG, which has been shown to reduce DTB time and improve short-term mortality.^24^ ^,^ ^25^ Additionally, when combined with prehospital dual antiplatelet therapy, these measures may further improve long-term outcomes in STEMI patients.^26^

Our study also revealed an association between cath lab arrival-to-balloon time and one-year mortality following STEMI, indicating that each minute of delay in this interval may increase the mortality risk by 2% within the first year (adjusted HR 1.02) (Table 3). Although the effect size was small, this finding may be reasonable. In addition to pharmacologic treatment in the initial stages of STEMI, prompt restoration of blood flow in the occluded coronary vessels is critical. While efforts to reduce DTB time has been explored,^27^ research focused on decreasing the duration from cath lab arrival-to-balloon inflation remains limited. It is essential to consider various factors that may influence cath lab arrival-to-balloon time, including patient’s vascular condition, the experience of the cardiologist, equipment preparation and readiness, and cath lab staff availability.^28^ ^,^ ^29^ Ongoing research on developing new techniques and guiding catheters to reduce the time from needle insertion to balloon inflation or mortality is imperative.^30^ Healthcare facilities must assess and optimize these factors to ensure timely and effective delivery of care to patients undergoing PCI procedures for STEMI.

Apart from the DTB time intervals, age, triage acuity level, initial troponin-I levels, and a history of diabetes mellitus and hyperlipidemia were identified as independent predictors for one-year mortality in STEMI patients in our study. The adverse impact of age and diabetes mellitus on short- and long-term mortality in STEMI patients has been extensively documented.^11^ Notably, we observed an association between a history of hyperlipidemia and reduced risk of one-year mortality (adjusted HR, 0.31, *P* < .001 in both models) (Table 3). Similar findings were also reported in previous research.^31^

One possible explanation for this is how a medical history of hyperlipidemia was defined, based on patients’ prior medical records and whether they received lipid-lowering therapy, primarily statins.^31^ In the Taiwan National Insurance program, the prescription of statins requires a confirmed diagnosis of hyperlipidemia. Therefore, we speculate that the reduced mortality risk observed in patients with a history of hyperlipidemia may be because these STEMI patients were receiving lipid-lowering therapy. Previous studies have found that patients with STEMI who were triaged as having lower acuity levels when they arrived at an ED experienced delays in ECG acquisition and reperfusion therapy.^17^ ^,^ ^32^ However, after adjusting for DTB time intervals, our study found that lower triage acuity was actually associated with a lower risk of one-year mortality. This may be because patient with higher triage acuity (triage level 1) often present with unstable vital signs, which are associated with a higher risk of mortality.

Wanamaker et al investigated the relationship between troponin levels at presentation and in-hospital mortality in STEMI patients undergoing PCI. They demonstrated that in-hospital mortality increases with elevated troponin levels at presentation, irrespective of baseline clinical risk.^18^ Our findings also revealed that initial troponin level is an independent predictor for one-year mortality in STEMI patients. Therefore, troponin levels in the early phase of STEMI may offer valuable long-term prognostic information in patients undergoing primary PCI.

## LIMITATIONS

Our study has several limitations. Firstly, it is a single-center study with a small sample size, potentially limiting its generalizability to other populations. Secondly, being retrospective in nature, there is a possibility of unmeasured confounders and selection bias that could have affected the results. Thirdly, the study covers a nine-year period, during which changes in hospital staff, policies, and guidelines may have introduced confounding factors. However, our sensitivity analysis, which controlled for the years of patient recruitment, yielded similar results. Nevertheless, further multicenter, prospective studies are warranted to validate our findings.

## CONCLUSION

Within the DTB interval, the time from door-to-ECG completion is crucial for one-year survival after STEMI, while cath lab arrival-to-balloon inflation may also be relevant. Strategies for improving long-term outcomes for STEMI patients should prioritize reducing the time from door-to-ECG acquisition. This could be attributed to the facilitation of early initiation of pharmacologic treatments, such as dual antiplatelet and anticoagulation therapy, in the initial stages of STEMI preceding PCI.

## Supplementary Information





