# Supplementary material for: Critical Time Intervals in Door-to-Balloon Time Linked to One-Year Mortality in ST-Elevation Myocardial Infarction
Source: West J Emerg Med. 2025 Jan 30;26(2):180–90. doi: 10.5811/westjem.20779 (PMC11931711; doi:10.5811/westjem.20779)
Supplement: Supplementary file 1 [file wjem-26-180-s001.docx]

**Supplementary Figure. 1.** Timeline of a STEMI patient requiring PCI from door to balloon inflation in the emergency department.


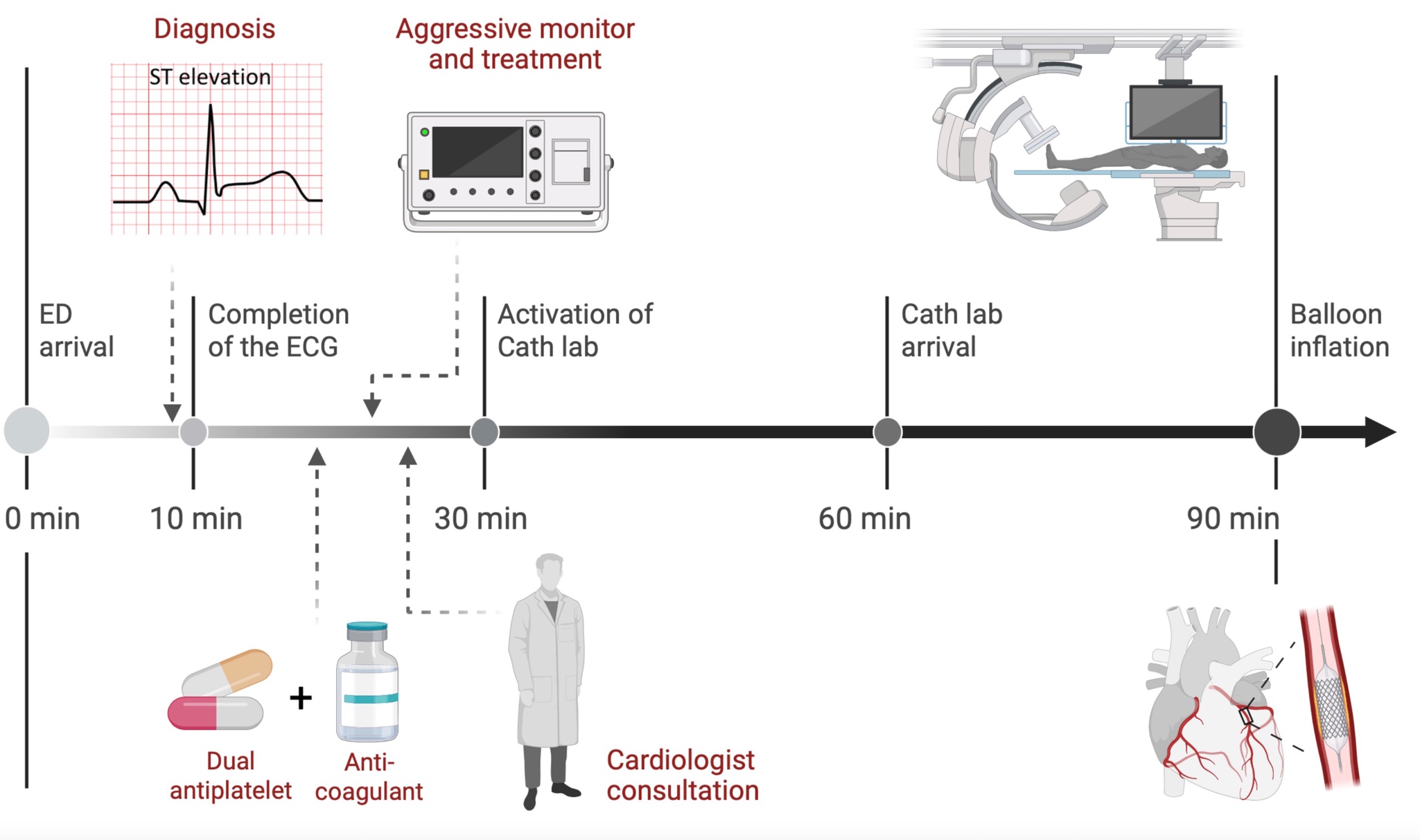


**Supplementary Table 1.** Sensitivity analysis for different time intervals within the DTB and short-term and long-term mortality outcomes.

|  | **In-hospital mortality** | | | | | **30-day mortality** | | | | | | | **1-year mortality** | | | | | |
| --- | --- | --- | --- | --- | --- | --- | --- | --- | --- | --- | --- | --- | --- | --- | --- | --- | --- | --- |
|  | **aOR** | **95% CI** | | ***P* value** | | **aOR** | | **95% CI** | | ***P* value** | | **aOR** | | | **95% CI** | | ***P* value** | |
| **Time interval was analyzed as dichotomized variable** | | |  | |  | |  | |  | |  | | |  | |  | |  |
| Door-to-ECG > 10 min | 3.61 | (1.41–9.27) | | 0.008 | | 2.72 | | (1.06–6.99) | | 0.038 | | 4.49 | | | (1.95–10.36) | | <0.001 | |
| ECG-to-activation > 20 min | 0.62 | (0.22–1.76) | | 0.364 | | 1.19 | | (0.49–2.88) | | 0.699 | | 1.67 | | | (0.80–3.50) | | 0.174 | |
| Activation-to-Cath lab-arrival > 30 min | 0.77 | (0.34–1.75) | | 0.529 | | 0.73 | | (0.33–1.61) | | 0.433 | | 0.63 | | | (0.31–1.31) | | 0.218 | |
| Cath lab-arrival-to-balloon time > 30 min | 1.46 | (0.69–3.08) | | 0.320 | | 1.47 | | (0.71–3.02) | | 0.299 | | 1.52 | | | (0.80–2.89) | | 0.200 | |
| **Time interval was analyzed as continous variable** | | |  | |  | |  | |  | |  | | |  | |  | |  |
| Door-to-ECG (min) | 1.06 | (1.01–1.11) | | 0.021 | | 1.05 | | (1.00–1.11) | | 0.034 | | 1.05 | | | (1.01–1.08) | | 0.006 | |
| ECG-to-activation (min) | 1.00 | (0.97–1.02) | | 0.797 | | 1.00 | | (0.98–1.02) | | 0.928 | | 1.00 | | | (0.99–1.02) | | 0.546 | |
| Activation-to-Cath lab-arrival (min) | 0.99 | (0.97–1.03) | | 0.758 | | 1.00 | | (0.97–1.03) | | 0.875 | | 1.00 | | | (0.98–1.03) | | 0.761 | |
| Cath lab-arrival-to-balloon (min) | 1.01 | (0.99–1.04) | | 0.227 | | 1.01 | | (0.99–1.03) | | 0.399 | | 1.03 | | | (1.00–1.05) | | 0.019 | |

Forward stepwise selection logistic regression was conducted with adjusted covariates including age, sex, ambulance-transported patient, triage level, troponin-I, diabetes, hypertension, hyperlipidemia, cerebrovascular accident, and chronic kidney disease.

aOR: adjusted odds ratio; CI: confidence interval; ECG: electrocardiography.

**Supplementary Table 2.** Sensitivity analysis of multivariable Cox models for predicting 1-year mortality after STEMI admission with the year of recruitment included in the models.

| Characteristics | Model 1 Adjusted HR (95% CI) | P value | | Model 2 Adjusted HR (95% CI) | | P value | |  |
| --- | --- | --- | --- | --- | --- | --- | --- | --- |
| Age (year) | 1.06 (1.04–1.09) | <0.001 | | 1.06 (1.04–1.09) | | <0.001 | |  |
| Triage level |  |  | |  | |  | |  |
| 1 | Reference |  | | Reference | |  | |  |
| 2 | 0.37 (0.20–0.68) | 0.002 | | 0.36 (0.20–0.67) | | 0.001 | |  |
| 3 | 0.13 (0.03–0.58) | 0.008 | | 0.12 (0.02–0.63) | | 0.012 | |  |
| Troponin-I (ng/mL) | 1.00 (1.00–1.01) | 0.09 | | 1.01 (1.00–1.01) | | 0.027 | |  |
| Diabetes mellitus | 1.55 (0.89–2.67) | 0.118 | | 1.64 (0.95–2.82) | | 0.075 | |  |
| Hyperlipidemia | 0.31 (0.17–0.57) | <0.001 | | 0.31 (0.17–0.56) | | <0.001 | |  |
| Year of patient recruitment |  |  | |  | |  | |  |
| 2013 | Reference |  | | Reference | |  | |  |
| 2014 | 0.63 (0.25–1.58) | 0.325 | | 0.59 (0.23–1.54) | | 0.283 | |  |
| 2015 | 1.05 (0.40–2.71) | 0.925 | | 0.93 (0.36–2.41) | | 0.883 | |  |
| 2016 | 0.39 (0.12–1.20) | 0.100 | | 0.36 (0.12–1.10) | | 0.074 | |  |
| 2017 | 0.51 (0.17–1.52) | 0.228 | | 0.50 (0.17–1.49) | | 0.213 | |  |
| 2018 | 0.28 (0.09–0.91) | 0.034 | | 0.27 (0.08–0.88) | | 0.003 | |  |
| 2019 | 0.51 (0.18–1.42) | 0.196 | | 0.48 (0.17–1.31) | | 0.150 | |  |
| 2020 | 0.65 (0.23–1.88) | 0.430 | | 0.63 (0.22–1.81) | | 0.395 | |  |
| 2021 | 0.27 (0.06–1.28) | 0.100 | | 0.27 (0.06–1.24) | | 0.092 | |  |
| Time interval |  |  | |  | |  | |  |
| Door-to-ECG > 10 min | 2.51 (1.22–5.14) | 0.012 | |  | |  | |  |
| Cath lab arrival-to-balloon > 30 min | 1.28 (0.73–2.27) | 0.390 | |  | |  | |  |
| Time interval |  |  | |  | |  | |  |
| Door-to-ECG time (min) |  |  | | 1.03 (1.00–1.06) | | 0.092 | |  |
| Cath lab arrival-to-balloon time (min) | | |  | | 1.02 (1.00–1.04) | | 0.037 | |

ECG: electrocardiography; HR: hazard ratio; STEMI: ST-segment elevation myocardial infarction.

The characteristics displayed in the table constitute the final variables that were included into the Cox models.

In the model 1, the time intervals were analyzed as dichotomized variables. In the model 2, the time intervals were analyzed as continuous variables.
